# Supplementary material for: Male germ cells support long-term propagation of Zika virus
Source: Nat Commun. 2018 May 29;9:2090. doi: 10.1038/s41467-018-04444-w (PMC5974187; doi:10.1038/s41467-018-04444-w)
Supplement: Supplementary file 1 — Supplementary Information [file 41467_2018_4444_MOESM1_ESM.pdf]

Supplementary Figure 1

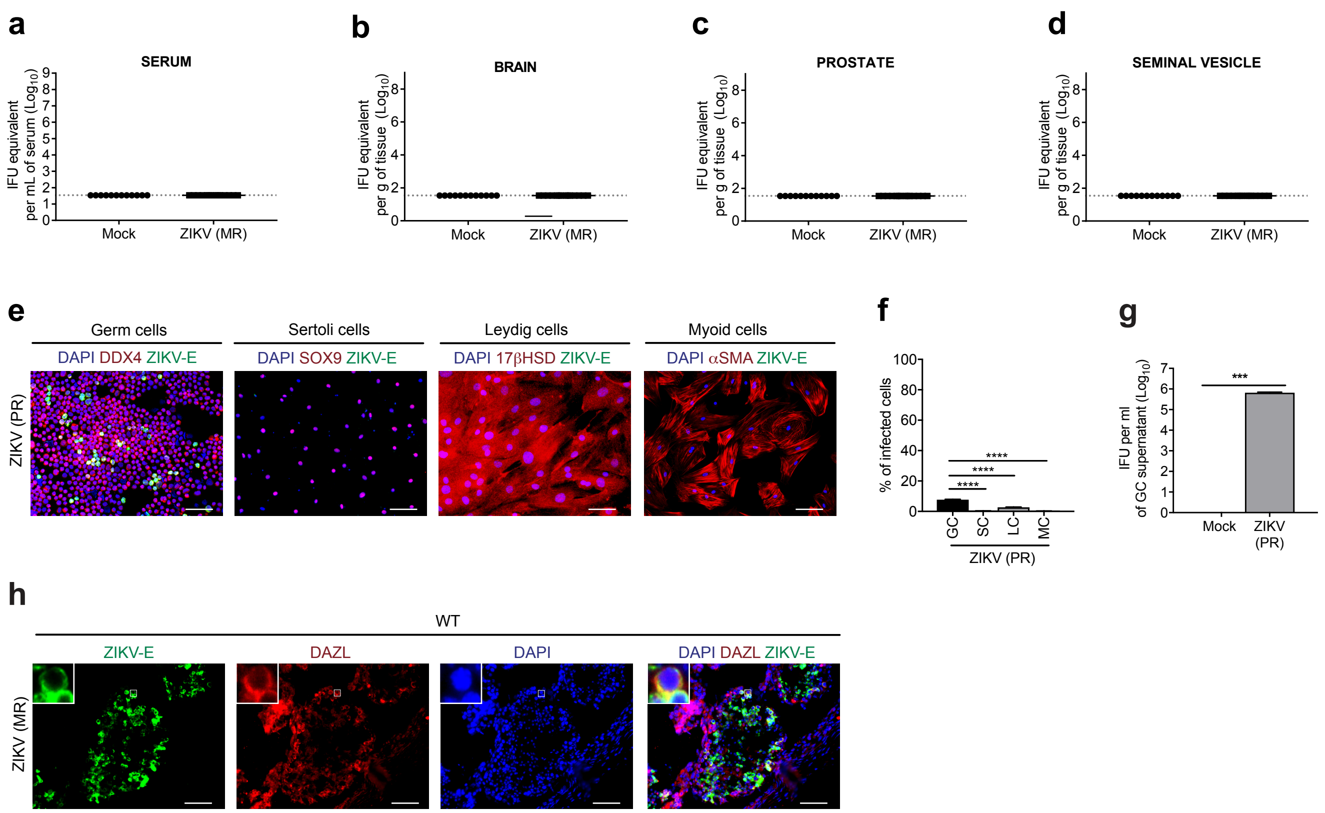

Supplementary Fig. 1 Murine male GC exhibit increased susceptibility to ZIKV infection. (**a-d**) qRT-PCR analysis of ZIKV vRNA levels in the serum (**a**), brain (**b**), prostate (**c**), and seminal vesicle (**d**) of ZIKV-infected (IFU = 1 x 10^8) wild type CD-1 mice at 60 dpi (n = 12 for mock- and 14 for ZIKV-infected). The dashed line represents the limit of detection. (**e-f**) ZIKV- (PRVABC59, MOI = 0.1 PFU $cell^{-1}$) infected GC, SC, LC, and MC immunostaining (**e**) and quantification (n = 5, 3, 3, and 8, respectively) (**f**) at 72 hpi. (**g**) Quantification of infectious ZIKV in supernatant of mock- and ZIKV- (PRVABC59, MOI = 0.1 PFU $cell^{-1}$) infected GC at 72 hpi by intracellular flow cytometry-based Vero assay (n = 3). (**h**) Immunostaining of ZIKV-infected (IFU = 1 x 10^5) wild type C57/BL6J testis with spermatocyte marker DAZL at 6 dpi. Statistical values are presented as mean ± s.e.m. and analyzed by two-sided *t*-test and one-way ANOVA, $***p \leq 0.001$, or $****p \leq 0.0001$. Scale bar, 50 µm. ZIKV strain is MR 766 throughout unless otherwise indicated.

Supplementary Figure 2

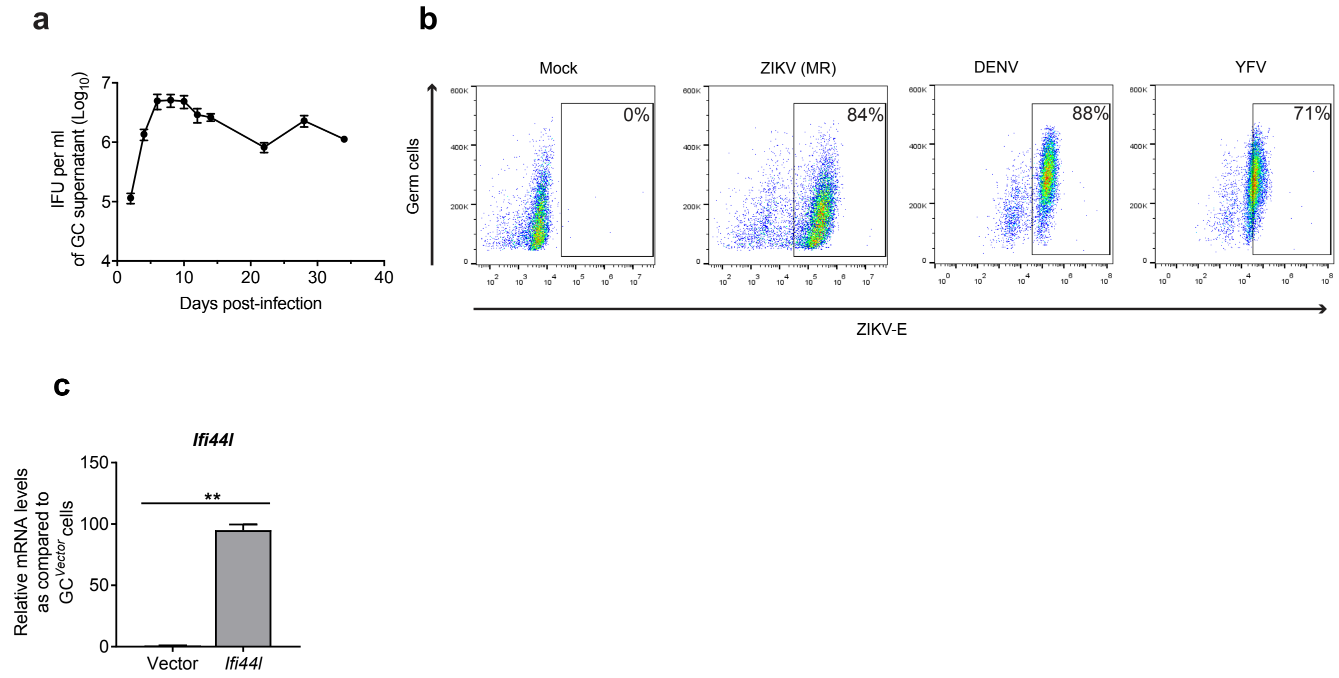

Supplementary Fig. 2 Long-term propagation of ZIKV by GC.
(**a**) Assessment of long-term propagation of ZIKV (PRVABC59) in GC up to 34 dpi with an intracellular flow cytometry-based Vero assay. (**b**) Intracellular flow cytometry analysis of GC infected with mock, ZIKV (MR 766), DENV, and YFV (MOI = 0.1 PFU $cell^{-1}$ for all) at 72 hpi. (**c**) qRT-PCR analysis of *Ifi44l* mRNA levels in GC^{Ifi44l} and GC^{Vector}. n = 3 for all experiments. Statistical values are presented as mean ± s.e.m. and analyzed by two-sided *t*-test, $**p \leq 0.01$.

Supplementary Figure 3

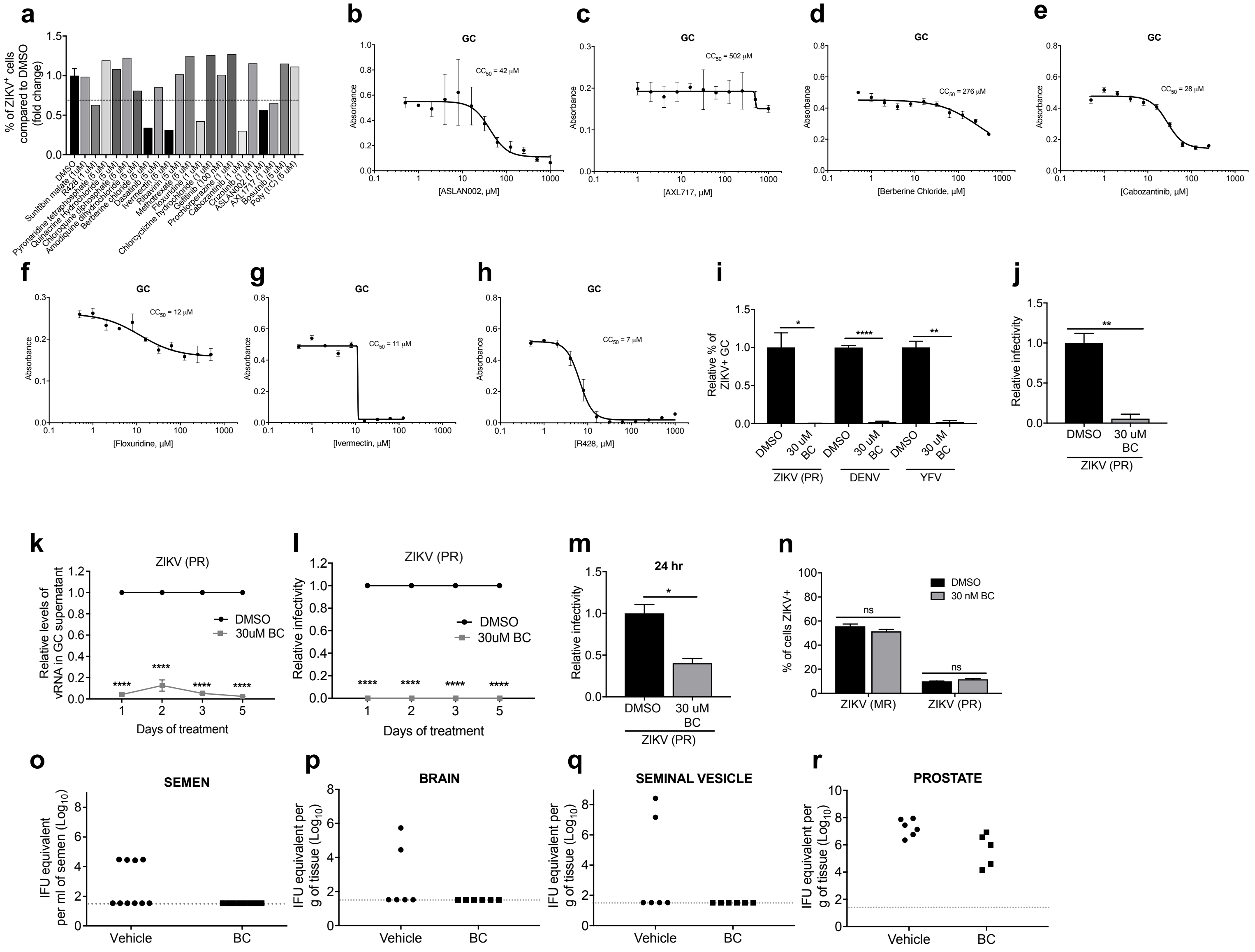

Supplementary Fig. 3 BC inhibits ZIKV infection of murine male GC *in vitro* and *in vivo*. (**a**) Small-molecule screen to identify compound(s) that inhibit ZIKV infection in cultured GC as assessed by intracellular flow cytometry. (**b-h**) Assessment of cytotoxicity of the 7 hit compounds in Fig. 5a using the MTS cell-based titer assay. (**i**) Fold change in percent of infection in GC by ZIKV (PRVABC59), DENV, and YFV (MOI = 0.1 PFU $cell^{-1}$ for all) in the presence of BC, as assessed by intracellular flow cytometry. (**j**) Infectivity of supernatant from ZIKV-infected (PRVABC59) GC in the presense of BC as assessed by intracellular flow cytometry-based Vero assay. (**k-l**) BC significantly reduced ZIKV (PRVABC59) infection in long-term infected GC. Culture media containing BC were refreshed every 24 h (starting at 35 dpi) and assessed for relative ZIKV vRNA in the supernatant by qRT-PCR (**k**) and for infectivity with Vero assay (l). (**m**) Infectivity of ZIKV (PRVABC59) after incubation with 30 μM BC for 24 h, measured by Vero assay. Samples were diluted 1:1,000. (**n**) Infectivity of ZIKV MR 766 and PRVABC59 on Vero cells in the presence of 30 nM BC. (**o-r**) qRT-PCR analysis of ZIKV vRNA levels in semen (**o**), brain (**p**), seminal vesicle (**q**), and prostate (**r**) of BC- and vehicle-treated ZIKV-infected mice at 6 dpi (n = 10 for vehicle-treated and 8 for BC-treated for semen; n = 6 for vehicle- and BC-treated for brain, seminal vesicle, and prostate). (a, i-m) Data are normalized to values obtained from DMSO-treated groups. n = 3 for all *in vitro* experiments unless otherwise indicated. Statistical values are presented as mean ± s.e.m. and analyzed by two-sided *t*-test, $*p \leq 0.05$, $**p \leq 0.01$, $****p \leq 0.0001$, or no significance (ns).

Supplementary Figure 4

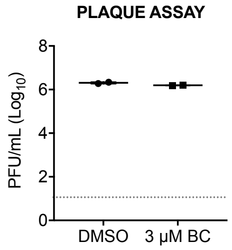

Supplementary Fig. 4 Analysis of plaque assay assessing the infectivity of ZIKV on Huh-7.5 cells in the presence of 3 μM BC or DMSO.
Statistical values are presented as mean ± s.e.m. and analyzed by two-sided *t*-test. ZIKV strain is MR 766.

Supplementary Table 1. Compounds used in candidate drug repurposing screen to inhibit ZIKV infection in GC.

Compounds	Known Biological Functions	Concentration Used
Berberine Chloride[1-3]	Antineoplastic, radiosensitizing, anti-inflammatory, anti-lipidemic, antidiabetic, antimicrobial	5 µM
Sunitinib Malate[4]	Tyrosine kinase receptor inhibitor	1 µM
R428[5]	AXL inhibitor	1 µM
Pyronaridine Tetraphosphate [6]	Antimalarial agent	5 µM
Quinacrine Hydrochloride[7]	Antimalarial agent	5 µM
Chloroquine diphosphate[8]	Antimalarial agent	5 µM
Amodiquine dihydrochloride[9]	Non-competitive inhibitor of histamine N-methyl transferase, antimalarial, and anti-inflammatory agent	5 µM
Dasatinib[10] [11]	c-Src protein kinase inhibitor	5 µM
Ivermectin[12] [1]	Antiviral activity against YFV, DENV-2 and WNV and a weak inhibitor of DENV protease	5 µM
Ribavirin[13]	Antiviral activity against hepatitis C	5 µM
Methotrexate[14]	Antimetabolites and immune system suppressant	5 µM
Floxuridine[14]	Antimetabolites and pyrimidine analog	1 µM
Chlorcyclizine hydrochloride[15]	Antihistamine	1 µM
Gefitinib[16]	EGFR inhibitor	100 nM
Prochlorperazine[17]	Dopamine (D_2) receptor antagonist	1 µM
Cabozantinib[18]	AXL inhibitor	1 µM
Crizotinib [19]	ALK and ROS1 inhibitor	1 µM
ASLAN002[20]	AXL inhibitor	1 µM
AXL1717[21]	AXL inhibitor	1 µM
Bosutnib[22]	AXL inhibitor	5 µM
Poly (I:C) [23]	Synthetic analog of double-stranded RNA	5 µM

Supplementary Table 2. Sequences of primers used for qPCR.

Gene	Primer Sequence
Mouse *Ifi44l*	F: AGCCAGGTTCAGAATGTCCAA<br>R: TCATAGGCTCCAACCTTCGC
Mouse *Ccl2*	F: CTGTCATGCTTCTGG GCCTG<br>R: CTGTCATGCTTCTGGGCCTG
Mouse *Ifr1*	F: AAGGATGCCTGTCTGTTCCG<br>R: TGCTTTGTATCGGCCTGTGT
Mouse *Ifit3*	F: TTCCCGGTTGACCTCACTCA<br>R: CTGAACTGCTCAGCCCAC
Mouse *Cxcl10*	F: CCAAGTGCTGCCGTCATTTTC<br>R:GGCTCGCAGGGATGATTTCAA
ZIKV (+) vRNA	RT: TACTTGTACAGCTCGTCCATGCCACTAACGTTCTTTTGCAGACAT<br>F: CCGCTGCCCAACACAAG<br>R: TACTTGTACAGCTCGTCCATG<br>Probe: 5'-/56- FAM/AGCCTACCT/ZEN/TGACAAGCAATCAGACACTCAA/3IABkFQ/- 3'
ZIKV (-) vRNA	RT: AACAGCCACAACGTCTATATCCCGCTGCCCAACACAAG<br>F: AACAGCCACAACGTCTATATC<br>R: CCACTAACGTTCTTTTGCAGACAT<br>Probe: 5'-/56 FAM/TTGAGTGTC/ZEN/TGATTGCTTGTCAAGGTAGGCT/3IABkFQ/- 3'
Mouse *Actb*	RT: CTGGATGGCTACGTACATGC<br>F: ATGCCGGAGCCGTTGTC<br>R: GCGAGCACAGCTTCTTTG<br>Probe: 5'-/5HEX/CCGCCACCA/ZEN/GTTCGCCATG/3IABkFQ/-3'
Human *Actb*	RT: CCTGGATAGCAACGTACATGG<br>F: CCTTGCACATGCCGGAG<br>R: ACAGAGCCTCGCCTTTG<br>Probe: 5'-/5HEX/TCATCCATG/ZEN/GTGAGCTGGCGG/3IABkFQ/-3'

References.

1 Varghese, F. S. *et al.* Discovery of berberine, abamectin and ivermectin as antivirals against chikungunya and other alphaviruses. *Antiviral Res* **126**, 117-124, doi:10.1016/j.antiviral.2015.12.012 (2016).

2 Varghese, F. S. *et al.* The Antiviral Alkaloid Berberine Reduces Chikungunya Virus-Induced Mitogen-Activated Protein Kinase Signaling. *J Virol* **90**, 9743-9757, doi:10.1128/JVI.01382-16 (2016).

3 Wen, S. Q., Jeyakkumar, P., Avula, S. R., Zhang, L. & Zhou, C. H. Discovery of novel berberine imidazoles as safe antimicrobial agents by down regulating ROS generation. *Bioorg Med Chem Lett* **26**, 2768-2773, doi:10.1016/j.bmcl.2016.04.070 (2016).

4 Bekerman, E. *et al.* Anticancer kinase inhibitors impair intracellular viral trafficking and exert broad-spectrum antiviral effects. *J Clin Invest* **127**, 1338-1352, doi:10.1172/JCI89857 (2017).

5 Holland, S. J. *et al.* R428, a selective small molecule inhibitor of Axl kinase, blocks tumor spread and prolongs survival in models of metastatic breast cancer. *Cancer Res* **70**, 1544-1554, doi:10.1158/0008-5472.CAN-09-2997 (2010).

6 Ekins, S. *et al.* Machine learning models identify molecules active against the Ebola virus in vitro. *F1000Res* **4**, 1091, doi:10.12688/f1000research.7217.3 (2015).

7 Bodenheimer, H. C., Jr. *et al.* Randomized controlled trial of quinacrine for the treatment of HBsAg-positive chronic hepatitis. *Hepatology* **3**, 936-938 (1983).

8 Farias, K. J., Machado, P. R. & da Fonseca, B. A. Chloroquine inhibits dengue virus type 2 replication in Vero cells but not in C6/36 cells. *ScientificWorldJournal* **2013**, 282734, doi:10.1155/2013/282734 (2013).

9 Boonyasuppayakorn, S., Reichert, E. D., Manzano, M., Nagarajan, K. & Padmanabhan, R. Amodiaquine, an antimalarial drug, inhibits dengue virus type 2 replication and infectivity. *Antiviral Res* **106**, 125-134, doi:10.1016/j.antiviral.2014.03.014 (2014).

10 Chu, J. J. & Yang, P. L. c-Src protein kinase inhibitors block assembly and maturation of dengue virus. *Proc Natl Acad Sci U S A* **104**, 3520-3525, doi:10.1073/pnas.0611681104 (2007).

11 de Wispelaere, M., LaCroix, A. J. & Yang, P. L. The small molecules AZD0530 and dasatinib inhibit dengue virus RNA replication via Fyn kinase. *J Virol* **87**, 7367-7381, doi:10.1128/JVI.00632-13 (2013).

12 Mastrangelo, E. *et al.* Ivermectin is a potent inhibitor of flavivirus replication specifically targeting NS3 helicase activity: new prospects for an old drug. *J Antimicrob Chemother* **67**, 1884-1894, doi:10.1093/jac/dks147 (2012).

13 Crance, J. M., Scaramozzino, N., Jouan, A. & Garin, D. Interferon, ribavirin, 6-azauridine and glycyrrhizin: antiviral compounds active against pathogenic flaviviruses. *Antiviral Res* **58**, 73-79 (2003).

14 Fischer, M. A. *et al.* Flaviviruses are sensitive to inhibition of thymidine synthesis pathways. *J Virol* **87**, 9411-9419, doi:10.1128/JVI.00101-13 (2013).

15 He, S. *et al.* Discovery, Optimization, and Characterization of Novel Chlorcyclizine Derivatives for the Treatment of Hepatitis C Virus Infection. *J Med Chem* **59**, 841-853, doi:10.1021/acs.jmedchem.5b00752 (2016).

16 Duran, A., Valero, N., Mosquera, J., Fuenmayor, E. & Alvarez-Mon, M. Gefitinib and pyrrolidine dithiocarbamate decrease viral replication and cytokine production in dengue virus infected human monocyte cultures. *Life Sci* **191**, 180-185, doi:10.1016/j.lfs.2017.10.027 (2017).

17 Simanjuntak, Y., Liang, J. J., Lee, Y. L. & Lin, Y. L. Repurposing of prochlorperazine for use against dengue virus infection. *J Infect Dis* **211**, 394-404, doi:10.1093/infdis/jiu377 (2015).

18 Zhou, L. *et al.* Targeting MET and AXL overcomes resistance to sunitinib therapy in renal cell carcinoma. *Oncogene* **35**, 2687-2697, doi:10.1038/onc.2015.343 (2016).

19 Jorge, S. E. *et al.* Responses to the multitargeted MET/ALK/ROS1 inhibitor crizotinib and co-occurring mutations in lung adenocarcinomas with MET amplification or MET exon 14 skipping mutation. *Lung Cancer* **90**, 369-374, doi:10.1016/j.lungcan.2015.10.028 (2015).

20 Okimoto, R. A. & Bivona, T. G. AXL receptor tyrosine kinase as a therapeutic target in NSCLC. *Lung Cancer (Auckl)* **6**, 27-34, doi:10.2147/LCTT.S60438 (2015).

21 Bergqvist, M. *et al.* Phase II randomized study of the IGF-1R pathway modulator AXL1717 compared to docetaxel in patients with previously treated, locally advanced or metastatic non-small cell lung cancer. *Acta Oncol* **56**, 441-447, doi:10.1080/0284186X.2016.1253866 (2017).

22 Ghosh, A. K. *et al.* The novel receptor tyrosine kinase Axl is constitutively active in B-cell chronic lymphocytic leukemia and acts as a docking site of nonreceptor kinases: implications for therapy. *Blood* **117**, 1928-1937, doi:10.1182/blood-2010-09-305649 (2011).

23 Li, Y. G. *et al.* Poly (I:C), an agonist of toll-like receptor-3, inhibits replication of the Chikungunya virus in BEAS-2B cells. *Virol J* **9**, 114, doi:10.1186/1743-422X-9-114 (2012).
